# An Eye-Tracking Paradigm for Analyzing the Processing Time of Sentences with Different Linguistic Complexities

**DOI:** 10.1371/journal.pone.0100186

**Published:** 2014-06-20

**Authors:** Dorothea Wendt, Thomas Brand, Birger Kollmeier

**Affiliations:** Medizinische Physik and Cluster of Excellence Hearing4all, Universität Oldenburg, Oldenburg, Germany; University of Pécs Medical School, Hungary

## Abstract

An eye-tracking paradigm was developed for use in audiology in order to enable online analysis of the speech comprehension process. This paradigm should be useful in assessing impediments in speech processing. In this paradigm, two scenes, a target picture and a competitor picture, were presented simultaneously with an aurally presented sentence that corresponded to the target picture. At the same time, eye fixations were recorded using an eye-tracking device. The effect of linguistic complexity on language processing time was assessed from eye fixation information by systematically varying linguistic complexity. This was achieved with a sentence corpus containing seven German sentence structures. A novel data analysis method computed the average tendency to fixate the target picture as a function of time during sentence processing. This allowed identification of the point in time at which the participant understood the sentence, referred to as the decision moment. Systematic differences in processing time were observed as a function of linguistic complexity. These differences in processing time may be used to assess the efficiency of cognitive processes involved in resolving linguistic complexity. Thus, the proposed method enables a temporal analysis of the speech comprehension process and has potential applications in speech audiology and psychoacoustics.

## Introduction

Speech intelligibility tests are an indispensable tool in clinical audiology. They can evaluate the consequence of sensory hearing loss (characterized by a frequency dependent hearing impairment) for the patient's communication abilities [1]–[4]. Beyond diagnostic applications, speech intelligibility tests are also often used to quantify the benefit of hearing aids or cochlear implants for individual patients. Typically, speech intelligibility tests measure the proportion of correctly repeated speech items, usually single words or single sentences [5]–[8]. However, research has shown that additional performance information about the ease of speech comprehension or cognitive effort during speech processing can complement traditional speech intelligibility measures. Increased cognitive effort is indicated by poorer task performance and processing time and can be measured in terms of recognition accuracy or reaction time, for instance [9], [10]. The current study focuses on developing a method for assessing the speech comprehension process and processing speed as indicators of the cognitive effort required at levels of high intelligibility. The proposed method is characterized by two main aspects: Firstly, a special speech corpus is applied that is optimized for both speech intelligibility measurements and controlled variation of linguistic complexity. Secondly, eye movements are tracked to provide an online assessment of speech processing during sentence comprehension. This study aims to determine whether this combination of speech intelligibility testing and eye tracking can detect a systematic deceleration in speech processing due to an increase in cognitive processing effort that is sufficiently large and robust to be used in audiology. A further question is whether the deceleration effect is detected by either recognition scores or reaction times alone.

### A. Speech intelligibility and linguistic complexity

Several studies reported that speech intelligibility is influenced by linguistic aspects of the speech material, such as context information, sentence structure, or level of complexity [11]–[13]. However, the role of linguistic aspects in speech comprehension, in particular in connection with hearing loss, has been largely neglected in standard audiological testing. In addition, speech intelligibility measurements provide little information about linguistic aspects in language comprehension, such as processing costs arising from different levels of cognitive load and/or linguistic complexity [13]. Recently, Uslar et al. [14] developed the Oldenburg Linguistically and Audiologically Controlled Sentences (OLACS) material to differentiate between acoustical and linguistic factors and their respective contributions to speech intelligibility measurement. Using the OLACS corpus, Uslar et al. measured speech reception thresholds (SRT) and reported a small effect of complexity on speech intelligibility (about 1–2 dB). However, studies in which participants were asked a comprehension question following sentence presentation revealed a stronger effect of linguistic complexity on sentence processing. For instance, Tun and colleagues [10] measured reaction times for sentences with different sentence structures presented at a clearly audible level. They observed reduced speech processing speeds for structures with higher linguistic complexity. It was argued that the reduced comprehension speed was caused by the increased cognitive processing demands of the more complex sentence structures. Hence, sentence complexity can lead to slower sentence processing. This suggests that sentence processing speed may be a more sensitive measure for detecting difficulties during sentence understanding than standard methods used in audiology, such as speech intelligibility tests. Reaction time, as reported by Tun et al. [10], and speech intelligibility measures are taken after the speech is presented. These offline measures do not provide any time-resolved information about the process of sentence comprehension, but instead reflect the end point of this process. On the other hand, an online analysis of processing time occurring *during* the presentation of the sentence is expected to provide a more direct measure of any temporal changes in speech processing that are not reflected by offline measures.

Another advantage of using response measures based on eye movements is their relative robustness against age effects [15]; latency and reaction times using a button press exhibit age-related differences [16]. This is an important issue when testing listeners with hearing impairment because hearing loss typically increases with age. For this reason, this study recorded both eye fixation and reaction time derived from pressing buttons.

### B. Analysis of eye movements with respect to speech processing

Eye movements are frequently used in psycholinguistic research in order to better understand how people process spoken sentences and to investigate linguistic aspects during sentence processing. A temporal relationship between speech processing and eye movements was shown in the pioneering study by Cooper [17], and confirmed in more recent studies (see [18] for a review). The visual world paradigm [19]–[21] was introduced in psycholinguistics to reveal the interaction between language and vision. In that paradigm, eye movements were recorded while simultaneously presenting spoken language and a visual scene that typically included the objects mentioned in the presented speech. Participants spontaneously fixated on the object that corresponded to the acoustical input. Several subsequent studies have investigated how and when the linguistic and visual information are integrated [22]–[29]. These recorded data were often used to investigate how linguistic processes determine the participants' sentence processing and understanding.

The method of analyzing the recorded eye-tracking data in the visual world paradigm, however, depends on the research question [18] and has not been adapted for use in audiology or made available to answer the research questions of the current study. For these reasons, an approach was adapted which combined several techniques from other (visual world) studies. The new approach was designed to meet the following requirements: a) the eye-tracking data must have a high temporal resolution; b) the test design must be symmetric, averaging out any systematic eye movement strategies, such as a preference for analyzing the pictures from left to right; c) the eye-tracking data analysis should shed light on speech comprehension and the decision process. Since the combination of these processing techniques is novel, the motivation behind each step is outlined in the following.

To investigate the effect of linguistic aspects on the comprehension process, the speech stimuli (words or sentences) were subdivided into separate time windows, as in previous studies [22], [26]. Due to the nature of speech, these segments varied slightly in duration. For this reason, a time alignment was applied. This allowed temporal averaging across segments and a high temporal resolution on a sub-segment basis.

As in previous visual world studies, the visual stimulus was subdivided into regions of interest (ROIs): one for the target picture and one for the competitor picture. Previous studies have analyzed whether these ROIs differ in their likelihoods of being fixated during each time segment [30], [31], or whether a ROI is looked at earlier in an experimental condition than in a control condition [22], [23]. Accordingly, the current study analyzed fixation rate as a function of time for different ROIs. Previous studies found that one region of interest was more likely to be fixated even before stimulus presentation, and emphasized that these baseline effects should be taken into account when analyzing the eye-tracking data [32]. However, methods that account for baseline effects have not often been applied in visual world studies. Therefore, the current study proposes a method that calculates the rates of fixations towards a target picture (in the current study a picture that matches the spoken sentence) in relation to the rate of fixation towards a competitor picture. As this is done both for target pictures on the left and on the right side, any systematic eye movement strategy that the participant uses, such as gazing preferably from left to right, is averaged out from the data. This is referred to as *symmetrizing* in the following. The applicability of assessing differences between fixations towards a target and a competitor was previously shown by other studies [33], [34]. A post-processing step is proposed that includes a bootstrap method to calculate the 95% confidence interval of the estimated probability that the participant fixates the target picture. Bootstrapping is an appropriate method for analyzing measurement statistics in situations where observed values violate normality or are unknown [35], [36]. In order to obtain a defined measure of processing speed and to detect the point in time when the target is recognized by the participant, a fixed threshold criterion is used, as described by McMurray and colleagues [37], [38].

The underlying hypothesis of this study is that the proposed eye-tracking paradigm can detect significant and robust reductions in sentence processing speed for sentence structures with increased linguistic complexity. This would qualify the proposed method for use in audiology. An increase in processing time, indicated by eye fixations as well as by reaction times, is then interpreted as evidence for a greater cognitive processing effort during sentence comprehension. This study had three main goals:

- Introduction of an eye-tracking paradigm that is adapted to the OLACS speech intelligibility test and enables online analysis of the time course of the sentence comprehension process for use in audiology.
- Introduction of a time-resolved statistical analysis technique for eye-tracking data that derives the decision moment (DM), defined as the point in time when the target is recognized by the participant. The analysis should take into account any systematic eye movement strategy employed by the participants.
- Evaluation of this paradigm and provision of normative data testing listeners with normal hearing in quiet.
- Identification of those sentence structures that show the most significant effects of linguistic complexity. As a prerequisite for a time-efficient clinical application, a reduced subset of test sentences will be needed for testing speech processing in listeners with hearing impairment in quiet and in noise.

## Material and Methods

### A. Participants

Seventeen volunteer participants (ten male and seven female) with normal hearing took part in the experiment. Hearing thresholds were measured at octave frequencies from 125 Hz to 8000 Hz. All participants had hearing thresholds less than 15 dB above normal threshold according to DIN EN ISO 8253-1 for all frequencies. All participants were native German speakers between 18 and 30 years of age (average age: 26 years) and either had uncorrected vision or wore corrective eyewear (glasses or contact lenses) when necessary.

### B. Ethics statement

Written consent was obtained from each participant prior to the experiments. The experiments were approved by the local ethics committee of the University of Oldenburg.

### C. Stimuli

#### Speech material

A total of 148 sentences from the OLACS corpus were used ([14]; a subset of the OLACS corpus can be obtained at http://www.aulin.uni-oldenburg.de/49349.html). Each sentence corresponded to one of seven different syntactic structures; there were approximately 21 sentences of each structure. The seven syntactic sentence structures fall into two major groups: verb-second structures and relative-clause structures (Table 1). Both groups contain sentences with canonical (subject-before-object) and non-canonical (object-before-subject) word orders.

**Table 1:** The Oldenburg Linguistically and Audiologically Controlled Sentences (OLACS).

Verb-second structures							
SVO	Der	kleine	Junge*_PTD_*	grüβt	den	lieben	Vater.
	The*_nom_*	little*_nom_*	boy*_nom_*	greets*_3sg_*	the*_acc_*	nice*_acc_*	father.
	*The little boy greets the nice father.*						
	Den	lieben	Vater*_PTD_*	grüβt	der	kleine	Junge.
OVS	The*_acc_*	nice*_acc_*	father	greets*_3sg_*	the*_nom_*	little*_nom_*	boy*_nom_*.
	*It is the nice father that the little boy is greeting.*						
	Die	liebe	Königin	grüβt	der*_PTD_*	kleine	Junge.
ambOVS	The*_amb_*	nice*_amb_*	queen*_fem,amb_*	greets*_3sg_*	the*_nom_*	little*_nom_*	boy*_nom_*.
	*It is the nice queen that the little boy is greeting.*						

Relative-clause structures									
SR	Der	Bauer,	der _PTD_			die	Ärztinnen	fängt,	lacht.
	The*_nom,sg_*	farmer*_nom,sg_*	who*_nom,sg_*			the*_nom_*	doctors_amb,*pl,fem*_	catches*_3sg_*	laughs*_3sg_*.
	*The farmer who is catching the doctors is laughing.*								
	Der	Bauer,	den _PTD_			die	Ärztinnen	fangen,	lacht.
OR	The*_nom,sg_*	farmer*_nom,sg_*	who*_acc, sg_*			the	doctors*_amb,pl,fem_*	catch*_3pl_*	laugh*_3sg_.*
	*The farmer whom the doctors are catching is laughing.*								
	Die	Bäuerinnen,	die		die		Ärztin	fangen*_PTD_*,	lachen.
ambSR	The*_amb_*	farmers*_amb,pl,fem_*	who*_amb_*		the*_amb_*		doctor*_sg,fem_*	catch*_3pl_*	laugh*_3pl_*.
	*The farmers who are catching the doctor are laughing.*								
	Die	Bäuerinnen,		die		die	Ärztin	fängt*_PTD_*,	lachen.
ambOR	The*_amb_*	farmers*_amb,pl,fem_*		who*_amb_*		the*_amb_*	doctor_amb,*sg, fem*_	catches*_3sg_*	laugh*_3pl_*.
	*The farmers whom the doctor is catching are laughing.*								

Example sentences for the seven sentence structures of the OLACS corpus. The disambiguating word from which the target picture could theoretically first be identified by the participant is indicated with *PTD* (point of target disambiguation). *Nom* (nominative), *acc* (accusative), and *amb* (ambiguous case) indicate the relevant case markings. *Sg* indicates singular forms and *pl* indicates plural forms. Verbs are either in their third person singular (3*sg*) or third person plural (3*pl*) form. *fem* indicates feminine nouns. *SVO*, *OVS*, and *ambOVS* sentence structures belong to the verb-second structures since they have either a subject-verb-object or an object-verb-subject sentence structure. *SR, OR, ambSR*, and *ambOR* sentence structures belong to the relative-clause structures. An English translation of the meaning of each example sentence is given in italics.

The group of verb-second structures includes three sentence structures: subject-verb-object (SVO), object-verb-subject (OVS), and ambiguous object-verb-subject (ambOVS). The SVO structure has the canonical word order for simple main clauses in German and is considered syntactically simple and easy to process [39]. The OVS structure is more complex because of its non-canonical word order [40]. The SVO and OVS structures are unambiguous with respect to their meaning and to the grammatical role of the sentence components (see Table 1). For example, the grammatical function of the first noun phrase is clearly marked for both the SVO structure (*Der kleine Junge_PTD_*, ‘The *_nom_* little*_nom_* boy*_nom_*’ *nom* indicates the nominative case marking) and the OVS structure (*Den lieben Vater_PTD_*, ‘The*_acc_* nice*_acc_* father’ *acc* indicates the accusative case marking). In both of these sentence structures, the disambiguating word, which is the word that clarifies the agent/object role assignment, is the first noun. For instance, the noun, *Junge_PTD_* ‘boy*_nom_*’ in the SVO sentence disambiguates the sentence in such a way that participants are theoretically able to relate the spoken sentence to the target picture as soon as the noun is spoken. In all cases, the onset of the word that disambiguates subject and object is termed the point of target disambiguation (PTD). Thus, the PTD was defined as the onset of the word that first enabled correct recognition of the target picture. Note that we chose the onset of the word even though in some sentence structures the recognition of the target was only made possible by the suffix of the disambiguating word. This was necessary because it was not possible to determine the exact point in time at which the disambiguation occurs during the spoken word.

The third verb-second structure, ambOVS, has an object-before-subject structure with a later point of disambiguation. In these sentences, the first article is ambiguously marked for case: the first article, *Die* (‘The*_amb_*’ *amb* indicates the ambiguous case marking; see Table 1) could indicate either subject or object function (and subsequently agent or object role) and only the article of the second noun, *der_PTD_* (‘the*_nom_*’ *nom* indicates the nominative case marking; see Table 1) is unambiguously case-marked.

The second group of sentence structures, which have relative-clause structures, includes four different structures of embedded relative clauses (Table 1): subject-relative (SR) clauses and object-relative (OR) clauses, with a PTD at the first relative pronoun *der_PTD_* (‘who*_nom,sg_*’) or *den_PTD_* (‘who*_acc,sg_*’ *sg* indicates singular form; see Table 1); and ambiguous subject-relative clauses (ambSR) and ambiguous object-relative clauses (ambOR) with a late PTD. The ambSR and ambOR sentence structures are disambiguated by the verb, *fangen_PTD_* (‘catch*_3pl_*’ *3pl* indicates the third person plural form) or *fängt_PTD_* (‘catches*_3sg_*’), of the embedded clause (Table 1).

The speech material provides different levels of linguistic complexity by varying three different structural factors of the sentence material: word order, embedding, and ambiguity. The preferred, canonical word order in German, like many other languages, is subject-before-object [41], [42]. The non-canonical object-before-subject word order is considered syntactically more complex [43] and has been shown to increase processing costs in the form of reduced accuracy and longer reaction times [9], [10], [44]. Another factor leading to increased processing costs is embedded relative-clauses [45], [46]. Within the relative-clause structures, processing costs can be further increased by word order [41], [46] (SR and OR structures in Table 1). The OLACS corpus further includes temporally ambiguous sentence structures, in which disambiguation occurs later. The ambiguity of these sentence structures (ambOVS, ambSR, ambOR) can lead to temporary uncertainty with regard to the grammatical role of the sentence components [14], [47]. Because of this ambiguity, the participant has to reanalyze the initial subject after the point of disambiguation. Hence, the ambiguity can lead to both increased processing cost and temporary misinterpretation of the sentence.

#### Visual stimuli

**Figure 1:**
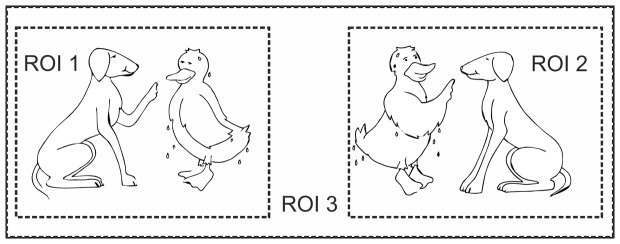
The visual stimulus. Example picture set for a sentence of the ambOVS sentence structure: *Die nasse Ente tadelt der treue Hund*. (The wet duck (acc.) reprimands the loyal dog (nom.), which means, “It is the wet duck that is reprimanded by the loyal dog”). A picture set consists of two single pictures. The dashed lines indicate the three regions of interest (ROI) and are not visible for the participants. ROI1 is the target picture and can be located on the left or right side of the picture set. ROI2 is the competitor picture. ROI3 is the background.

In total, picture sets for 150 sentences of the OLACS corpus were created. Each picture set consisted of two pictures (Figure 1). One of the two pictures, the target picture, illustrated the situation described by the sentence. In the competitor picture, the roles of agent (the active character) and object (the passive character) were interchanged. In each picture, the agent was always shown on the left side in order to facilitate fast comprehension of the depicted scene. Presenting both pictures at the same time ensured that participants did not assign agent and object roles using only visual information. All of the figures illustrated in the picture sets had the same size in order to avoid effects of contrast between the figures. Care was taken in selecting actions, agents, and objects that were non-stereotypical, such that the action was not characteristic for the agent (for example, baking is a typical action of a baker). This constraint was employed to make sure that participants did not make premature role assignments based on any anticipation of an agent's characteristic action. The picture set was divided into three regions of interest (ROI): ROI1 defined the target picture, ROI2 the competitor picture, and ROI3 defined the background. The target picture was shown randomly either on the left or right side of the computer screen. Consequently, the positions of ROI1 and ROI2 were not fixed, but changed randomly from trial to trial.

#### Validation of the visual stimuli

To ensure that both pictures in a particular picture set could be parsed and interpreted equally well, a subset of the graphical material was tested by measuring the reaction times of 20 participants. For 106 picture sets, the reaction time for each picture was measured (212 single pictures). For that purpose, each sentence was presented visually in written form on a computer screen for 1500 ms. Afterward one picture, either the target or the competitor picture, was shown on the computer screen, and the participants had to decide whether the presented picture matched the previously displayed sentence. Participants were instructed to respond as quickly as possible and reaction times were measured. Note that the sentences were simplified for the validation of the visual stimuli: the modified sentences all had a subject-verb-object structure, and the adjectives of the verb-second structures and the matrix verbs of the relative-clause structures were omitted in this pre-test. For instance, Figure 1 shows the picture corresponding to the example sentence, “The dog reprimands the duck.” By modifying the sentences to have the same syntactical structure, any effects of linguistic complexity on reaction times were avoided. The statistical significance of the differences in reaction times for the two pictures of one set was calculated for all participants using a paired t-test with a 5% significance level. If a significant difference was found, the picture set was excluded from the eye-tracking study. Of the 106 picture sets tested, two sets were excluded. Because so few picture sets had to be excluded, no formal reaction time validation was performed for the additional 44 picture sets that were produced later and added to the experimental set. Thus, in total, 148 different picture sets were used for the eye-tracking experiment.

### D. Procedure

For the experiments, an OLACS picture set was presented visually on a computer screen while the recorded sentence was presented via headphones. First, the participants performed one training block, which contained all 148 picture sets. After training, six test blocks, containing 110 sentences each, were performed. In total, each participant listened to 660 sentences. 148 sentences were presented in quiet at a level of 65 dB SPL. Two conditions with different background noises were employed for a different study: 444 sentences were presented in different noise conditions. These 592 sentences were randomly distributed across the six test blocks. In order to avoid retrieval strategies, 68 filler trials were presented across all test blocks (11–12 filler trials per test block). During a filler trial, either the target or the competitor picture was depicted on both sides of the screen, with the positions of the agent and object reversed in one of the two pictures. Therefore, either both of the pictures matched the spoken sentence or neither did. These trials forced the participants to fixate on both pictures.

The visual stimulus was presented 1000 ms before the onset of the acoustic stimulus. Participants were instructed to identify the picture that matched the acoustic stimulus by pressing one of three keys as quickly as possible: The “A” indicated that participants assigned the target to the left picture, and “L” indicated assignment to the right picture; participants were instructed to press the space bar if they were not able to clearly assign one target picture to the spoken sentence. The position of the selected keys enabled the participants to leave their hands on the keyboard during the experiment so they did not have to look at the keyboard to search for the right key. After each trial, participants were asked to look at a marker at the center of the screen so that a drift correction could be performed. At the beginning of each test block a calibration was done using a nine-point fixation stimulus. The completion of one test block of trials took about 20 min. After each block, participants had a ten-minute break. The entire measurement took about three hours per participant, which was divided into two sessions.

### E. Apparatus

An eye-tracker system (EyeLink 1000 desktop system including the EyeLink CL high-speed camera, SR Research Ltd.) was used to monitor participants' eye fixations with a sampling rate of 1000 Hz. The pictures were presented on a 22″ multi-scan color computer screen with a resolution of 1680×1050 pixels. Participants were seated 60 cm from the computer screen. A chin rest was used to stabilize the participant's head. Although, viewing was binocular, the eye-tracker sampled only from the dominant eye. Auditory signals were presented via closed headphones (Sennheiser HDA 200) that were free-field compensated according to DIN EN ISO 389-8 (2004). For the calibration of the speech signals, a Brüel & Kjær (B&K) 4153 artificial ear, a B&K 4134 1/2 inch microphone, a B&K 2669 preamplifier, and a B&K 2610 measuring amplifier were used. All experiments took place in a sound-insulated booth.

## Data Analysis

### A. Time alignment

Since the sentences differed in length, a time alignment was employed to allow comparisons across sentences. This was realized by dividing each trial into six segments, as shown in Table 2. Note that the choice of segment borders and the evaluation of eye-tracking data during these segments were selected to best fit the employed OLACS speech material. Knoeferle and colleagues [26] showed that for German sentences with an initially ambiguous structure, sentence interpretation happens immediately after the point in time at which the combination of visual and linguistic information disambiguates the sentence. Therefore, segment borders were defined according to the word that first enabled correct recognition of the target picture. Segment 1 corresponds to the time from the onset of the visual stimulus until the onset of the acoustical stimulus. The spoken sentence was presented during segments 2 through 5. The time from the end of the spoken sentence until the participant responded by pressing the response key was denoted as segment 6. The segment borders and the corresponding points in time (in ms) during the eye-tracking recordings were determined for each sentence and averaged over all sentences of a single sentence structure (see Table 2).

**Table 2:** Time segments used for time alignment across sentences.

	Segment 1	Segment 2	Segment 3	Segment 4	Segment 5	Segment 6
Segment borders/sample	0–100	100–200	200–300	300–400	400–500	500–end
Verb-second structure	*no acoustic stimulus*	Der kleine	Junge	grüβt den	lieben Vater.	*response time*
		The little	boy	greets the	nice father.	
Mean segment borders/ms	0–1000	1000–1745 (±130)	1745–2340 (±135)	2340–2995 (±130)	2995–4140 (±151)	4140–end (±114)
Relative-clause structure	*no acoustic stimulus*	Der Bauer,	der die Ärtzinnen	fängt,	lacht.	*response time*
		The farmer	who the doctors	catches	smiles.	
Mean segment borders/ms	0–1000	1000–1885 (±200)	1885–2755 (±136)	2755–3430 (±131)	3430–4450 (±143)	4450–end (±238)

Time segments for the verb-second and relative-clause structures used for time alignment across sentences. The first row gives the borders of each segment in time samples. Segment 1 describes the time from the onset of the measurement until the onset of the acoustical stimulus. The spoken sentence was presented during segments 2 through 5. Segment 6 corresponds to the time between the end of the spoken sentence and the participant's response. An example sentence is given for each group. The mean segment borders (in milliseconds) were calculated over all sentences in the group after the resampling procedure (± standard deviation).

### B. Calculation of the target detection amplitude (TDA)

**Figure 2:**
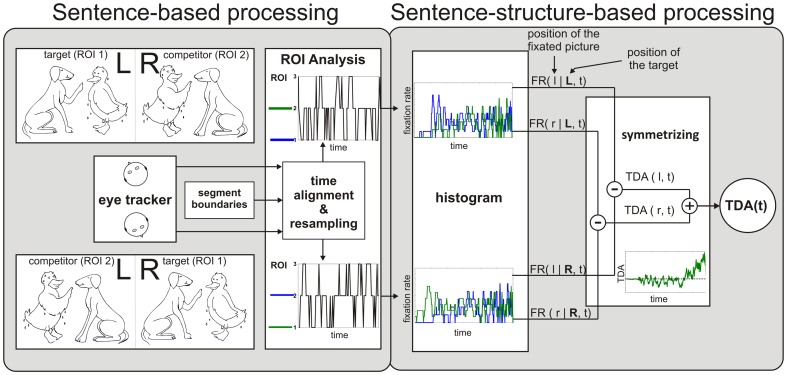
Schematic diagram for the analysis of the recorded eye fixation data. The first two stages of the calculation of the target detection amplitude (TDA) are depicted, namely the sentence-based processing and the sentence-structure-based processing stages.

**Figure 3:**
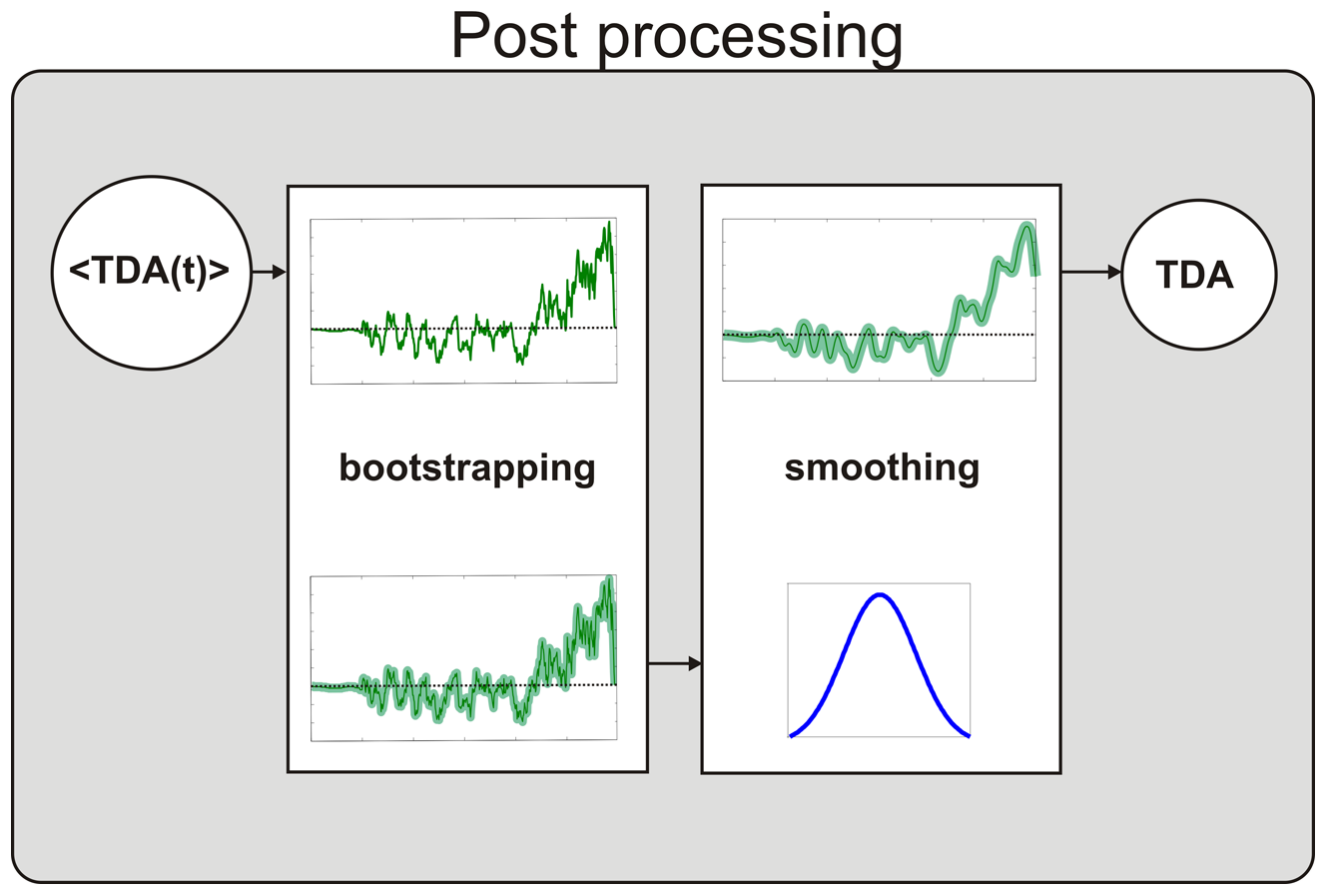
Post-processing stage of the analysis of the recorded eye-fixation data. Post processing of the target detection amplitude (TDA), including the bootstrap method and Gaussian smoothing.

**Figure 4:**
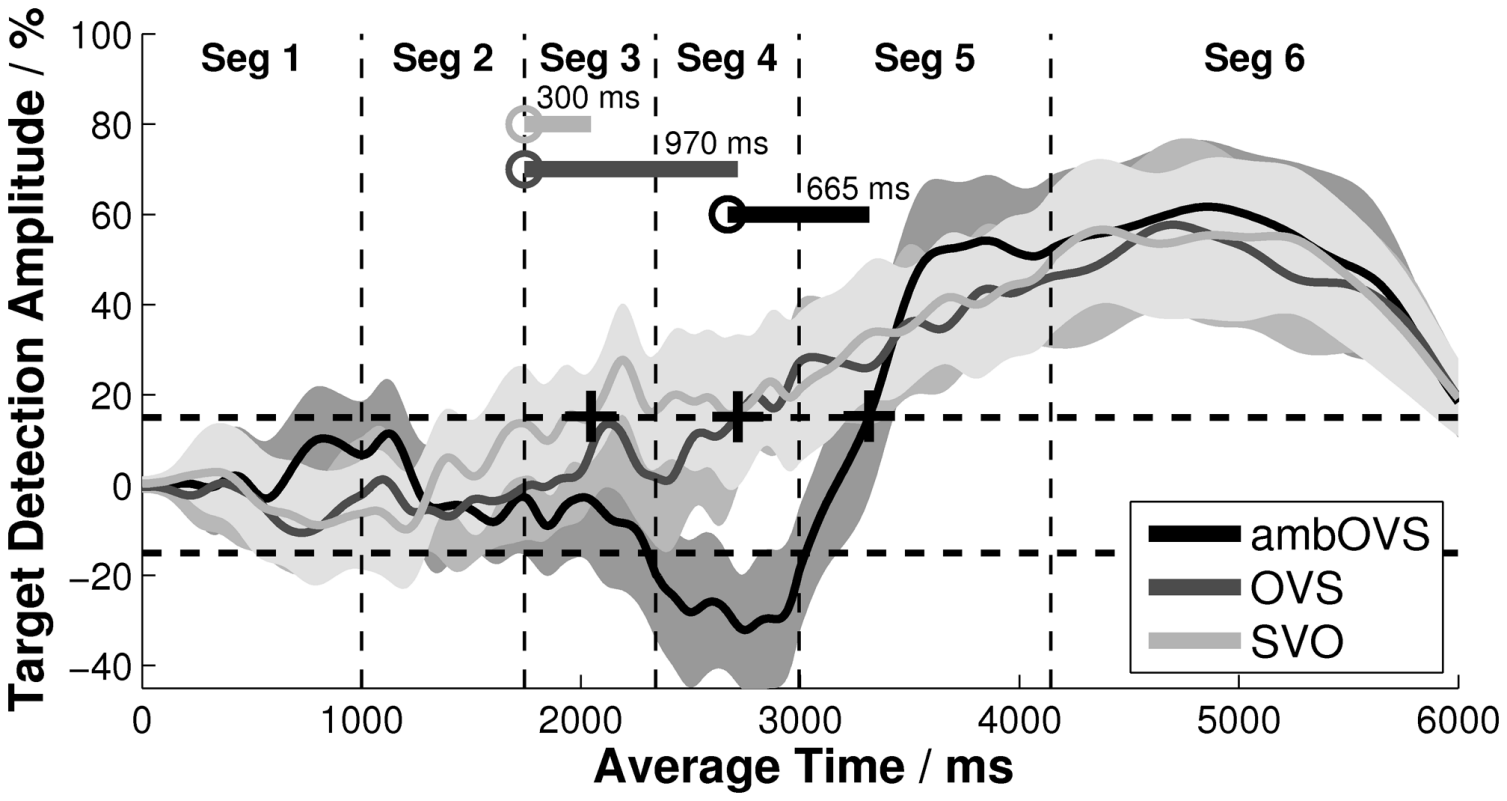
Mean target detection amplitude (TDA) for the verb-second structures. Mean target detection amplitude (TDA) averaged over all participants for the verb-second structures, i.e. the subject-verb-object (SVO), object-verb-subject (OVS), and the ambiguous object-verb-subject (ambOVS) structures. The shaded areas illustrate the 95% confidence intervals for each individual curve. The + signs at 2045 ms, 2715 ms, and 3315 ms denote the decision moments (DM) where the TDA first exceeded the threshold (15% of the TDA). The circles denote the point of target disambiguation (PTD): at 1745 ms for the SVO and OVS sentences and at 2650 ms for the ambOVS sentences. The horizontal lines denote the disambiguation to decision delay (DDD), which is the distance between the PTD and the DM.

**Figure 5:**
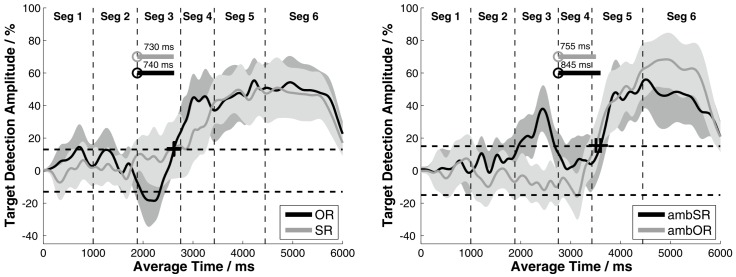
Mean target detection amplitude (TDA) for the relative-clause structures. Mean target detection amplitude (TDA) averaged over all participants for the relative-clause structures of the OLACS. The shaded areas illustrate the 95% confidence intervals for each curve. Left panel: unambiguous subject-relative clause (SR) vs. unambiguous object-relative clause (OR) sentences; DMs (+) at 2615 ms and 2625 ms, respectively. Right panel: ambiguous subject-relative clause (ambSR) vs. ambiguous object-relative clause (ambOR) sentences; DMs (+) at 3600 ms and 3510 ms, respectively. Circles denote the points of target disambiguation (PTD): at 1885 ms for the SR and OR sentences and at 2755 ms for the ambSR and ambOR sentences.

The eye-tracking data were used to calculate the target detection amplitude (TDA). The TDA quantifies the tendency of the participant to fixate on the target picture in the presence of the competitor picture. The data analysis for the TDA was divided into three stages (Figures 2 and 3). In the first stage, the calculation was sentence based (left panel in Figure 2). The recorded eye-tracking data were analyzed and the fixations on the target (ROI1), the competitor (ROI2), and the background (ROI3) were calculated as functions of time. Trials in which the target was presented on the left side were considered separately from those in which the target was on the right. A time alignment and a resampling stage were employed to associate the observed fixations of the ROIs with the appropriate sentence segment (see Table 2). To synchronize the segment borders across sentences, the first five segments were individually rescaled to a fixed length of 100 samples using an interpolation algorithm. The length of segment 6 depended on the mean reaction time of the participant, with a maximal length of 200 samples (see Table 2). For instance, if the reaction time was 1500 ms, the last segment was rescaled to a length of 150 samples. For reaction times longer than 2000 ms, the signal was cut to a length of 200 samples. This was done because 1000 ms after the offset of the sentence, on average, participants fixated less frequently on the target picture (as can be seen in segment 6 in Figure 4 and Figure 5). This may have been because no more information could be gained after this time. The segment-based resampling used a fixed number of samples per segment (except for the last segment), which resulted in a segment-dependent sampling rate depending on the individual length of each segment. This resampling not only allowed comparison across sentences of one structure, but also across different sentence structures.

The second stage of the TDA calculation was sentence-structure based (Figure 2). For a given (interpolated) time sample, the fixated ROIs were averaged across all sentences of a given sentence structure, resulting in an average fixation rate (right panel in Figure 2). Further analysis of the data showed that the fixation rates of the background (ROI3) did not differ significantly between sentence structures. Since this study examines the differences in the time courses of the TDAs for different sentence structures, the fixation rates of the background (ROI3) were not considered in the calculation of the TDA. Thus, the fixation rates of target (ROI1) and competitor (ROI2) did not add up to 100%. Only trials in which the participants selected the correct picture were used for further analysis. This selection was done in order to analyze time patterns of eye fixations that reflected the dynamics of the recognition process for correctly identified sentences only.

#### Symmetrizing

In general, participants tended to fixate more frequently on the left picture. This effect was independent of the position of the target picture and was most noticeable in segment 1, before the acoustical stimulus was presented. This tendency towards the left picture probably arose from the usual reading direction and was exploited in the paradigm by always presenting the agent of each scene on the left side of each picture (except in filler trials). This agent-left convention supported the participant in systematic and fast analysis of each picture as uncertainties about the agent's and the object's roles within each picture were reduced. The agent-left convention may have supported the listeners' left-to-right strategy. To correct for this, the test design was symmetrized: in random order, the target picture was presented equally often on the left and right sides. Subsequently, the fixation rate was averaged across all trials, averaging out any left-to-right picture reading strategy. One half was subtracted from the resulting averaged target fixation rate (which ranges between 0 and 1) in order to center it around 0. The result was then multiplied by 2. This resulted in the TDA, which assumed the value -1 for sole fixations of the competitor, 0 for random fixation, and 1 for sole fixations of the target. The calculation of the TDA was split into different processing steps, which allowed analysis of the fixation rates for left and right targets separately. Four different fixation rates FR(s|S, t) were considered, with s denoting the position of the fixated picture (with l for left side and r for right side), S denoting the position of the target picture (with L for left side and R for right side), and t denoting the time. Depending on the position of the target, the two fixation rates of the competitor pictures FR(r|L, t) and FR(l|R, t) were subtracted from the respective fixation rates of the target pictures FR(l|L, t) and FR(r|R, t). This gave the TDA for the left picture: 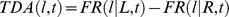
and for the right picture:
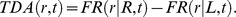



The position-independent total TDA was expressed using the sum of the two side-dependent TDA(s,t):
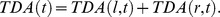



The total TDA(t) was a function of time and quantified the tendency to fixate on the target picture within the arrangement of alternative pictures. Positive values indicated more fixations on the target picture and negative values indicated more fixations on the competitor picture. A value near zero reflected the inability to differentiate between the two pictures at a given point in time. The TDA(t) was computed for all 17 participants, resulting in a set *M* of 17 values for each sentence structure at a given point in time t:



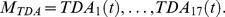



### C. Post-processing

To compute the time-smoothed mean value and estimate the 95% confidence interval of the TDA, this set was input to a post-processing stage, as depicted in Figure 3.

A bootstrapping resampling procedure was applied [35], [36] to estimate the mean value and 95% confidence interval of the average TDA across participants for the different OLACS sentence structures without assuming any underlying distribution. This type of bootstrapping procedure has been successfully applied before to analyze eye-tracking data [48]. This bootstrapping was necessary because the underlying distribution of the mean value across the set ***M***TDA at a given point in time was unknown and could vary across different sentence structures. For each time point, a sample from ***M***TDA was randomly selected with replacement 17 times and averaged to provide a random estimate of the mean value <TDA(t)> across participants. This process was repeated 10,000 times, resulting in a resampled data set containing 10,000 values that approximated the estimated distribution of <TDA(t)>. From this distribution, the 95% confidence intervals and the mean value <TDA(t)> were obtained. Finally, a Gaussian smoothing filter with a kernel size of 25 samples was applied in order to reduce the random fluctuations of the <TDA(t)>. The resulting signal was called TDA (see e.g. Figure 4)**.**

### D. Calculation of the decision moment (DM) and the disambiguation to decision delay (DDD)

The decision moment (DM) was defined as the point in time from which the mean TDA exceeded the 15% threshold for at least 200 ms. The threshold was chosen as 15% TDA because small fluctuations in the TDA are not relevant for the investigation of speech processing. The time between the PTD and the DM was calculated for each sentence structure and defined as disambiguation to decision delay (DDD). This DDD is interpreted as a measure of processing time: The greater the DDD, the longer the processing time and the slower the speed of sentence processing.

## Results and Discussion

### A. Picture recognition rates and reaction times

The picture recognition rates–the percentage of correctly identified target pictures (by pushing the correct button)–for each sentence structure (see Table 3) were averaged across all participants. Before conducting further analyses, picture recognition rates were transformed to rationalized arcsine units (rau) according to [49].

**Table 3:** **Picture recognition rates and reaction times.**

	Verb-second structures		
Sentence structure	Recognition rate/rau	Reaction time/ms	Decision moment/ms
SVO	97.6±5.1	2057±477	2045(Δt = 645)
OVS	105.8±8.1	1956±421	2715 (Δt = 1380)
ambOVS	81.0±4.3	1944±300	3315 (Δt = 275)

	Relative-clause structures		
Sentence structure	Recognition rate/rau	Reaction time/ms	Decision moment/ms
SR	101.4±8.8	2029±411	2615 (Δt = 1515)
OR	91.6±9.9	1965±447	2625 (Δt = 335)
ambSR	100.9±8.7	2084±643	3600 (Δt = 895)
ambOR	96.2±4.4	1898±367	3510 (Δt = 340)

Picture recognition rates and reaction times obtained from the keyboard responses, and the calculated decision moments (DM) for each sentence structure. The mean picture recognition rates in rationalized arcsine units (rau), reaction times (ms), and DMs (ms) were calculated over all participants for both verb-second and relative-clause structures of the OLACS corpus. The calculated DMs are listed for each sentence structure with the corresponding width Δt (in milliseconds) of the confidence interval at the 15% threshold along the timeline.

To investigate the effect of sentence structure on picture recognition, a one-way repeated measures ANOVA was conducted for both groups of sentence structures. The factor sentence structure was significant for both groups of sentence structures (verb-second: F(2;32) = 36.2, p<0.001; relative-clause: F(3;48) = 7.4, p<0.001). Multiple pairwise comparisons with Bonferroni correction revealed differences in picture recognition rates between the SVO and ambOVS structures (p<0.001), reflecting lower picture recognition rates for the ambOVS structure. The picture recognition rate for ambOVS sentences was lower than that for OVS sentences (p<0.001). For the relative-clause structures, the pairwise comparisons revealed significant differences between SR and OR structures (p = 0.001) and between OR and ambSR structures (p = 0.002).

In general, significantly lower picture recognition rates, in particular for the object-first sentence structures (ambOVS and OR structures) suggest that linguistic complexity affects picture recognition performance. This is not self-evident: all of the sentences were presented in quiet at a constant sound pressure level of 65 dB and were acoustically controlled for equal intelligibility (for detailed information, see [14]), so they should all have been equally understandable. For that reason, the differences in picture recognition rates found here are evidence that linguistic factors influence the processing of syntactically complex structures in combination with the visual stimuli.

The reaction times were measured offline: participants were asked to press the response button after the end of the sentence. To investigate the effect of sentence structure on reaction time, a one-way repeated-measures ANOVA was conducted for both groups of sentence structures. The factor sentence structure was not significant for either group, indicating that sentence complexity did not affect reaction time within this paradigm. Note that the offline measures, recognition rate and reaction time, did not follow the same pattern across sentence structures, suggesting different response strategies and criteria. However, this effect was not considered further because the online measures used in this paper took place markedly before the (offline) button press. In addition, only correct trials were considered for the online analysis.

### B. Eye-fixation data

The target detection amplitude (TDA) functions for the verb-second and relative-clause structures are depicted in Figures 4 and 5, respectively. The dashed vertical lines reflect the averaged segment borders. The time points corresponding to these segment borders are shown for both groups of sentence structures in Table 2. The dashed horizontal lines in Figures 4 and 5 indicate the thresholds of ±15% TDA. The decision moment (DM) is the point in time at which the TDA exceeded the threshold for at least 200 ms; it is indicated with a plus sign for each sentence structure. The DM was interpreted as the moment at which participants recognized the target, since they fixated the target picture significantly more freuquently than the competitor. The circles indicate the PTD corresponding with the words denoted in Table 1. The horizontal lines starting at the PTDs depict the disambiguation to decision delay (DDD).

#### Verb-second structures

Figure 4 shows the TDAs for the three sentence structures with verb-second structures. The TDAs fluctuated between the thresholds (±15% TDA) around zero during the first two segments for all three sentence structures: neither target nor competitor picture was fixated preferably. Since the PTDs for the two unambiguous sentence structures (SVO, OVS) did not occur until the beginning of segment 3, the DM was not expected before the beginning of segment 3. The fact that the TDA fluctuated around zero during the first segments indicated the success of the symmetrizing method in averaging out any systematic strategy of the participants. If the tendency of fixating the left picture first would not have been compensated for, the TDA would have differed significantly from zero.

The early case marking of the first noun phrase, *Der kleine Junge_PTD_* (‘The_nom_ little_nom_ boy_nom_;’ see Table 1), in the SVO structure allowed an early thematic role assignment, so participants were able to identify the noun phrase referent, *Junge_PTD_* (‘boy_nom_’) as the agent and to recognize the target even before the end of the spoken noun. This was indicated by an early DM during segment 3, with a DDD of 300 ms, for the SVO structure.

The first noun phrase, *Den lieben Vater_PTD_* (‘The_acc_ nice_acc_ father’ see Table 1), of the unambiguous OVS structure also provided role information at the very beginning of the spoken sentence. But despite the early PTD, the DM of the OVS structure was observed during segment 4, one segment after the first noun, *Vater_PTD_* (‘father’), was spoken. Thus, the DDD for the OVS structure was about 970 ms. So although the 95% confidence intervals of the SVO and OVS structures overlapped slightly at the DMs, their DDDs differed by more than 600 ms.

Object-first sentences with a late PTD, as in the ambOVS structure, had a markedly different TDA time course. The DM of the ambOVS structure occurred during segment 5, after the onset of the second article, *der_PTD_* (‘the_nom_’ Table 1), which disambiguated the sentence in segment 4. This resulted in a DDD of about 665 ms. Note that the DDD for the ambOVS structure was about 300 ms shorter than that of the unambiguous sentence structure, OVS. In addition, a strongly negative TDA was observed for the ambOVS structure at the end of segment 3, indicating that participants were preferentially fixating on the competitor picture. The negative TDAs were interpreted as a temporary misinterpretation arising out of listeners' preferences for subject-before-object word order. German shows a general preference of subject-before-object word order [41], [42]. So listeners expected a subject-before-object sentence structure and tended to interpret the first noun phase, *Die liebe Königin* (‘The*_amb_* nice*_amb_* queen*_fem, amb_*’ see Table 1), as the subject of the sentence. As a result, the competitor was fixated more frequently at the beginning of the sentence. This temporary misinterpretation only occurred before the sentence had been disambiguated by the article of the second noun phrase, *der_PTD_* (‘the*_nom_*’).

#### Relative-clause structures

The left panel of Figure 5 shows the average TDAs of the unambiguous relative-clause structures (SR and OR structures). For both structures, the TDAs fluctuated around zero during the first two segments, indicating that the target was not recognized. For both sentence structures, the case-marking relative pronoun, der_PTD_ (‘who_nom, sg_’) or den_PTD_ (‘who_acc, sg_’ see Table 1), of the embedded phrase disambiguated the sentence; this is indicated by the PTD at the very beginning of segment 3. The DMs of both sentence structures occurred at the end of segment 3 and the DDDs varied between 730 ms and 740 ms.

The right panel of Figure 5 shows the TDAs of the two ambiguous relative-clause structures (ambSR and ambOR). It is clear that the embedded verbs (*fangen_PTD_* ‘catch_3pl_’ and *fängt_PTD_* ‘catches_3sg_’ Table 1) resolved the roles of agent and object: the PTD was located at the beginning of segment 4. The DMs were observed in segment 5, with a DDD of 755 ms for the ambOR structure and 845 ms for the ambSR structure. Note that for the unambiguous structures, the first article of the embedded sentence (*der_PTD_* ‘who*_nom,sg_*’ or *den_PTD_* ‘who_acc,sg_’ see Table 1), which had an average length of about 135 ms, disambiguated the spoken sentence. In contrast, the disambiguating word for the ambiguous sentence structure was the embedded verb (*fangen_PTD_* ‘catch_3pl_’ and *fängt_PTD_* ‘catches_3sg_’ see Table 1), with an average length of about 575 ms. For most of these embedded verbs the disambiguating information about the agent/object role assignment was not given until the suffix. Since the PTD was defined as the onset of the disambiguating word, the different word lengths (135 ms vs. 575 ms) had to be accounted for when comparing the DDDs of the different relative-clause structures. After subtracting the length of the disambiguating word, the remaining DDD was much smaller for the ambiguous structures than for the unambiguous structures.

Participants were not expected to discriminate between the two pictures before the PTD, so the TDAs of the two sentence structures should not differ markedly before the PTD. Surprisingly, a significant positive TDA was observed for the ambSR structure shortly after the relative pronoun *die* (‘the_amb_’ see Table 1) in segment 3. If this unexpected early increase in the TDA had been caused by the participants' subject-first preference, then it should have also been reflected in the time course of the ambOR structure. For instance, if the plural form of the noun used for the ambiguous subject-relative and object-relative clauses had helped the participants to recognize the target earlier, this should have been indicated in the TDA of both sentence structures. It would have appeared as an early increase in the TDA for the ambSR structure and a decrease in the TDA for the ambOR structure. However, this was not the case: no significant decrease in the TDA was observed in segment 3 or at any later point in time.

There is some evidence that this unexpected effect was caused by the presence of more acoustical cues in the ambSR sentences. Carroll and Ruigendijk [46] pointed out that there was a small but significant difference in the speech rate between the words in segment 2 in the ambSR and ambOR structures. The participants may have used the slower speech of the ambSR sentences to differentiate between the two sentence structures even before the PTD was reached. However, further investigations are needed to identify the reason for the early increase. With the rationale of this study and an audiological application in view, the ambSR structure is not recommended for further studies using the eye-tracking paradigm.

### C. Precision of the estimated decision moment

In order to define the temporal precision of the DM, the temporal width Δt of the confidence interval of the TDA was determined at the DM (Table 3). That is, the width Δt of the confidence interval was calculated at the point in time at which the TDA began to exceed the 15% threshold for a period that lasted at least 200 ms. The width Δt varied from about 275 ms to 1515 ms across the seven different sentence structures. Sentence structures with a steep slope at the DM exhibited a small Δt. The steepest slopes were measured for the ambiguous sentence structures. While Δt was the smallest for the object-first sentences with ambiguous structures (ambOVS and ambOR; Δt<500 ms), for unambiguous subject-first sentence structures (SVO and SR) Δt showed high variability, due to the flat slope of the TDA at the DM. Possible differences in the process of recognizing the target between unambiguous and ambiguous sentence structures may have influenced the time course of the TDA and caused a smaller Δt for the ambiguous structures. Different decision-making processes are discussed in the following section.

## General Discussion

An eye-tracking paradigm was introduced with a time-resolved statistical data analysis technique that enabled online analysis of the time course of the sentence comprehension process. The main objective of this study was to evaluate the paradigm for a group of listeners with normal hearing using a speech intelligibility test that was audiologically controlled with respect to speech intelligibility and linguistic complexity. The novel data analysis technique was designed to detect time-dependent effects in speech comprehension at various levels of linguistic complexity even at high speech intelligibility levels. The technique was designed with a potential application in audiological research in mind.

An increase in processing time could indicate that people have trouble in everyday communication situations, since the speech rate can be about 140–180 words per minute in ordinary conversations [50]. A person who is slow at sentence processing may miss speech information later in the conversation because he/she is still processing a “backlog” of past sentences or words. This slower sentence processing is interpreted as an indicator of increased cognitive processing demands even at high speech intelligibility levels. Speech intelligibility tests, in which speech recognition performance is recorded sentence by sentence, failed to detect these increased processing demands at high intelligibility. In the long run, however, this slowing down and an increased processing effort may prevent people from participating in a conversation. So far, there is no established method in audiological research that allows this kind of online analysis of speech comprehension. The results reported in this study highlighted another important advantage of the online measure: misinterpretations could be detected while the speech was presented; offline measures of processing time may be insensitive to these difficulties in sentence comprehension since participants can overcome them before the sentence is completed.

### A. Effect of sentence structure on TDA and processing time

In general, processing time was expected to be increased for sentences with a higher level of linguistic complexity. Different levels of linguistic complexity were achieved using the OLACS material by altering word order, embedding relative clauses, and introducing ambiguity. In general, the results indicated that the DDD, which was interpreted as a measure of processing time, greatly depended on the sentence structure. Word order had a strong effect on sentence processing time for the verb-second structures. Longer processing times were found for the non-canonical compared to the canonical sentence structure, indicated by an increase in the DDD of almost 600 ms. An increase in processing time indicated additional cognitive processing costs, which were expected to arise from the non-canonical word order. Increased processing costs caused by non-canonical word order have been reported in many other psycholinguistics studies [39], [41], [44], [46]. As expected, sentence processing was slower for embedded structures: the DDD was 300 ms for the SVO structure and 730 ms for the SR structure. Interestingly, no increase in processing time was observed for the object-relative (OR) structure compared to the subject-relative (SR) structure. It is possible that the additional processing cost of the embedded sentence structure covered any smaller differences in processing time caused by changes in word order.

Several earlier studies already reported that sentence structure complexity caused processing difficulties, increasing the cognitive processing load during speech comprehension. This was revealed using different measures, such as reaction times, recognition scores, and pupil size [9], [10], [50]. Tun and colleagues [10] presented different sentences structures and examined participants' reaction times when answering comprehension questions. They reported an increase in reaction time for complex sentence structures, indicating an imposed cognitive processing effort due to linguistic complexity even at a high intelligibility level. Piquardo et al. [51] reported that pupil size increased significantly during storing and processing of complex object-before-subject sentence structures compared to syntactically less complex subject-before-object sentence structures. They interpreted the pupillary enlargement as an indicator of the engagement of cognitive effort during the processing of the complex sentences. However, a significant effect of sentence structure on pupil size could only be measured *after* the verbal presentation of the sentence. The results of the current study supported most of these findings, underscoring the validity of this paradigm. The DDD greatly depended on sentence structure: syntax-related difficulties during sentence processing were observed by measuring processing time. In contrast to measures such as reaction times or pupil size, used in the previously mentioned studies, the proposed eye-tracking paradigm taps into sentence processing while the sentence is being spoken. Early literature about the visual world paradigm reported that participants had difficulties *during* speech comprehension either on the sentence or the word processing level [e.g., 19, 21, 26]. The fact that sentence structure had no significant effect on offline reaction times (measured by participants' button press) in this paradigm strengthens the assertion that the proposed online measure of processing speed is more sensitive for detecting processing difficulties.

Processing was expected to be slower for ambiguous sentence structures than for unambiguous structures. Interestingly, this was not the case; instead, sentence processing time was actually smaller for the ambiguous sentence structures than for their unambiguous counterparts. This was particularly evident for the ambOVS structure. Furthermore, negative TDAs indicated more fixations towards the competitor picture and were interpreted as a temporary misinterpretation of the agent and object roles. Temporal processing difficulties have been reported by Knoeferle and colleagues using the visual world paradigm [26]. They assessed online the participants' processing difficulties that arose from their expectations of thematic roles in German SVO and OVS sentence structures. The negative TDA values in the current study indicate that the eye movements and the time curve of the TDA was influenced not only the speech signal but also by the listeners' preferences and expectations. Only after the PTD did the participants realize that they had identified the wrong picture as the target picture; they then had to adjust their decision and choose the other picture; this decision is indicated by a steep increase in the TDA. This temporary misinterpretation of the sentence led to a sudden acceleration in the decision-making process: the participant just had to choose the other picture. This may make processing faster than for unambiguous sentence structures, and is reflected in the smaller DDDs.

### B. Audiological application and further research

As discussed in the previous section, our results are largely consistent with other studies, especially in psycholinguistic research investigating linguistic aspects in sentence processing. Those studies did not address audiological aspects. Moreover, (psycho-) linguistic aspects of the speech material have been considered to a lesser extent in the audiological research field to date. The data presented demonstrate the value of the paradigm for assessing aspects of cognitive processing in a speech comprehension task. The paradigm presented here was developed as a combination of methods from both research fields: recording eye-fixation data during sentence processing, which is typically used in psycholinguistic studies, and using a sentence corpus that was developed for speech intelligibility measurements. This combination may provide a useful tool for diagnostic purposes in audiology.

Modern hearing aids offer several signal-processing technologies for adapting to different environments, depending on the type of hearing impairment. These include, for instance, dynamic range compression and noise reduction. Research concerning the benefit of hearing aid signal processing traditionally focused on the effects on speech recognition scores or intelligibility measures (such as the SRT). However, speech reception measures often lack the sensitivity to test the benefits of hearing aid algorithms or acclimatization effects of the user. One reason is that SRTs for standard speech intelligibly tests are typically at negative signal-to-noise ratios (SNRs). However some hearing aid algorithms, such as noise reduction algorithms, often require positive SNRs for optimum performance [52]–[54]. In this situation, speech intelligibility is high and speech intelligibility tests in audiology suffer from ceiling effects. In addition, several studies propose to focus less on improving speech intelligibility measures and more on the effort during speech processing [55], [56]. The effects of these signal-processing technologies on the effort required for speech understanding is still an active field of research.

In any case, the opportunity to assess the processing speed of hearing-impaired listeners would be a valuable tool for the individualization of hearing aid fitting. Individual processing efforts in speech perception have been tested using subjectively rated efforts. Brons et al. [55] investigated subjectively rated effort of participants for different hearing aids and the effect of their noise-reduction outputs on the effort. They showed that hearing aid settings influenced the effort involved in listening to speech in noise. In addition, they reported that effort may change between conditions for which speech intelligibility remains constant. Minimizing listening effort is a desirable goal for fitting and adjusting hearing devices and should be supported by an effective and objective way of testing processing effort in audiology. The standard measures and methods used in audiology do not provide an effective and objective way of testing sentence processing and processing effort. The proposed objective measure of processing speed may be used for the design, selection and fitting of hearing devices to the individual listener so that they can be adapted to the individual processing speed and/or processing effort in perceiving speech in acoustically difficult situations. Furthermore, the eye-tracking method introduced here is able to detect differences in processing time that arise from sentence complexity. This could also be relevant for diagnostic purposes, enabling differentiation between peripheral, sensorineural deficits in speech comprehension and more cognitive, centrally located deficits.

However, this study is only the first step towards the application of this paradigm in audiology. Note that the scope of this manuscript includes presenting the proposed method and evaluating it with the OLACS sentence corpus. A systematic study of the influence of bottom-up vs. top-down processing in background noise or hearing impairment is beyond the scope of this study, and several issues need to be clarified before the method can be broadly applied:

1. Further studies are needed to examine the interaction of sensory factors, such as hearing loss and masking noise, with the linguistic factors investigated in this study. By applying different noise types, the effect of energetic, modulation, and informational masking on speech processing and the required effort at controlled speech intelligibly levels should be investigated systematically. In addition, it has been shown that speech intelligibility can also be influenced by the rate of speech [57], so the sensitivity of the proposed paradigm to changes in speech rates is a relevant aspect that should be addressed in future studies.
2. To gain better insight into how individual factors, such as hearing loss, might affect processing speed, it is important to assess speech processing in individual participants. The results of the current study indicate that the TDA varied widely across participants. The confidence intervals shown here include both inter-individual and intra-individual test-retest variance. A more precise TDA time course and DM could be estimated for a single participant by increasing the number of sentences per sentence structure.
3. For clinical studies, it is important to have a relatively small number of trial repetitions, so the number of sentence structures tested should be reduced for this purpose. In general, the set of verb-second structures showed strong effects on processing speed in response to changes in word order. In contrast, the expected word order effects were not seen for the relative-clause sentence structures. Consequently, of the seven different sentence structures from the OLACS corpus, the verb-second structures are the most promising for analyzing processing time and are likely to be sufficient for audiological applications.

## Conclusions

This study developed and evaluated an eye-tracking paradigm that provides a time-resolved, online measure of sentence processing, revealing the influence of linguistic complexity. Experimental data from 17 participants with normal hearing tested in quiet showed that the proposed method was able to detect syntax-related delays during sentence processing using speech material that was optimized for use in audiology. As the results were in line with findings of other psycholinguistic studies, it can be concluded that the method proposed here is valid. Moreover, the experimental data showed that the proposed methods can be relevant with regard to audiological research:

1. The target detection amplitude (TDA) provides a statistically supported, time-resolved measure that directly reflects the participants' comprehension of the sentence. This measure can even be negative, which indicates a temporary misinterpretation of the presented sentence. This underlines the advantage of an online measure that provides information about the time course of speech processing.
2. The eye-tracking paradigm reveals effects of linguistic complexity on processing time that were not found in offline measures of processing speed, such as reaction time, assessed by pressing a button. Processing time was influenced by sentence structures in a systematic way, even though all measurements were performed at the same high level of intelligibility. This indicates that the proposed measure provides information about cognitive processes in speech understanding that go beyond classical speech intelligibility measures.
3. The highest contrast in processing time was observed for the SVO, OVS, and ambOVS sentence structures. Thus the verb-second structures provide a reasonable subset for practical applications, for example in audiology.

In conclusion, the paradigm presented here has a strong potential for use in audiology, where measures revealing differences in speech processing at high levels of intelligibility are highly desired.
